# Smoking behaviours and attitudes towards campus-wide tobacco control policies among staff and students: a cross-sectional survey at the University of Birmingham

**DOI:** 10.1186/s12889-020-8321-9

**Published:** 2020-02-19

**Authors:** Suzanne E. Bartington, Ruth Wootton, Philippa Hawkins, Amanda Farley, Laura L. Jones, Shamil Haroon

**Affiliations:** 1 Institute of Applied Health Research, University of Birmingham, Edgbaston, Birmingham, UK; 2 Wellbeing Services, University of Birmingham, Birmingham, UK; 3 Workplace Wellbeing, University of Birmingham, Birmingham, UK; 4 Institute of Applied Health Research, University of Birmingham, Edgbaston, Birmingham, UK

**Keywords:** Tobacco control, Smoke-free policy, Smoking behaviour, Smoking attitudes, Universities

## Abstract

**Background:**

Tobacco control policies have potential to be an effective strategy for the reduction of smoking prevalence and secondhand smoke (SHS) exposure in tertiary educational settings worldwide. The aims of this study were to collect baseline data among staff and students, to measure smoking behaviours and attitudes towards introduction of campus-wide tobacco control policies within a UK higher education setting.

**Methods:**

Cross-sectional study using data collected by web-based questionnaire administered to employed staff and enrolled students (undergraduate/postgraduate) at the University of Birmingham from May 2016 to April 2017. Information was obtained regarding demographic characteristics, tobacco usage patterns and attitudes towards a revised campus tobacco control policy using a 21-item survey tool. Logistic regression analyses were used to explore associations between participant characteristics and support for smoke-free or tobacco-free campus policy options, evaluated by crude and adjusted Odds Radios (OR) after controlling for confounding factors (significance level: *P* < 0.05).

**Results:**

A total of 934 survey responses were received, of whom 780 participants provided complete information on staff or student status and were included in the present analysis. Current smoking prevalence was 14% (*N* = 109; 95% confidence interval (CI) 11.6–16.6). Overall, 66.3% (95% CI: 62.9–69.7) of participants supported a smoke-free campus; 68.5% (95% CI: 65.2–71.8) endorsed restrictions for tobacco sales and just under half of respondents (47.3%; 95% CI: 43.8–50.9) supported a ban for electronic cigarettes/vaping device use on campus. Smoking status was an independent predictor of support for tobacco control, with the lowest level of support for a smoke-free campus among daily (adjusted OR 0.02; 95% CI: 0.01–0.05) and intermittent smokers (adjusted OR 0.06; 95% CI: 0.02–0.16).

**Conclusions:**

Overall, the majority of staff and students participating in this baseline survey supported implementation of a smoke-free or comprehensive tobacco-free campus policy. These findings may inform the development and future implementation of a revised tobacco control policy at the university which reflects contemporary attitudes and considers a broad range of implementation issues, including behaviour change and environmental adaptations.

## Background

Smoking is a major avoidable cause of preventable disease and premature mortality in the UK responsible for approximately 78,000 premature deaths each year [1]. The harmful effects of involuntary environmental exposure to secondhand smoke (SHS) are well established, with no safe level of exposure for human health [2]. Since ratification of the World Health Organization Framework Convention for Tobacco Control (WHO-FCTC) in 2005, [3] many countries worldwide have introduced smoke-free legislative policies providing protection from exposure to tobacco smoke in indoor workplaces and public places, including educational establishments. There is consistent evidence for a positive impact of smoking bans in public spaces for improved cardiovascular health outcomes, and reduced mortality for associated smoking-related illnesses achieved primarily through reduced SHS exposure [4, 5].

However, existing national UK smoke-free legalisation does not restrict smoking or use of tobacco products in outdoor public spaces or across postsecondary educational settings, where almost 50% of young people aged between 17 and 30 years participate in education and training in the UK [6]. This age cohort coincides with a recognised period of health behaviour transition, including change from intention to regular smoking [7]. Tobacco control policies in such settings have potential to deliver multiple public health benefits through protection of staff, students and visitors from SHS exposure, [8] prevention of smoking initiation and improved uptake of smoking cessation [9]. Furthermore, people who stop smoking before the age of 30 years avoid more than 90% of the lung cancer risk attributable to tobacco compared to those who continue to smoke [10]. Restrictions on outdoor smoking may also provide wider benefits including improved staff and student productivity, litter reduction, decreased fire risk and increased student retention [11]. Finally, influencing university students may be important for modifying social norms relating to smoking as many will become future opinion and thought leaders.

Voluntary campus-wide tobacco control policies may comprise a range of measures, in the United States context these have previously been defined as: (i) *smoke-free:* ban of smoking in all indoor and outdoor areas; (ii) *tobacco-free:* ban of smoking and smokeless tobacco product use in all indoor and outdoor areas, which may also be extended to include prohibition of all activities relating to tobacco promotion, sponsorship and sale, such as institutional disinvestment from tobacco companies and withdrawal of direct/indirect research funding strategies involving the tobacco industry [12]. In addition, smoke or tobacco-free policy measures may include specific provision for, or restrictions upon the use of electronic cigarettes or vaping devices. Such policies have gained increasing popularity for adoption among university and colleges worldwide, particularly in the United States (US) where in a 2018 national survey, over one third (35.2%) of US postsecondary institutions had adopted comprehensive tobacco-free policies, and 10.1% smoke-free policies respectively, with higher rates of adoption among public institutions [13].

Several studies have observed tobacco control policies implemented in such settings to be associated with significant reductions in smoking prevalence among university students, [14] reduced cigarette butt littering, [15] and a shift in social norms favouring smoke-free environments [16]; with stronger tobacco-free policies associated with reduced intention to smoke on campus [17]. The baseline level of support and engagement among staff and students has been recognised as a predictive factor for effective implementation, influencing both policy adoption [18, 19] and compliance [20]. However, there remains limited information regarding contemporary smoking patterns and levels of support for different tobacco control policies among staff and students at university and college campus settings in the UK.

In this context, we sought to identify smoking behaviours and attitudes among staff and students working or studying at the University of Birmingham. This baseline assessment comprised the first phase of an ongoing programme of research to inform development and future implementation of a revised university campus tobacco control policy. For the purpose of this study: we adopted the following definitions for outdoor campus areas (as a voluntary extension of existing smoke-free legislation for enclosed public spaces and workplaces): (i) *smoke-free campus policy* – ban of smoking, and; (ii) a *comprehensive tobacco-free campus policy* –ban of smoking, use of e-cigarettes and sales of tobacco products on campus. Our research objectives were to: (a) determine baseline patterns of tobacco usage and smoking behaviours; (b) investigate levels of support for smoke-free or comprehensive tobacco-free policy options; (c) identify independent predictive factors associated with support for a smoke-free or comprehensive tobacco-free control policy.

## Methods

### Study design

This was a cross-sectional, population-based study using baseline data obtained by self-administered online questionnaire developed using items adopted from the Global Adult Tobacco Survey [21] and the Health Survey for England (HSE) [22]. The survey instrument was pilot tested among 45 staff and students attending a University Wellbeing Event, to assess acceptability and feasibility, and subsequently modified prior to implementation. Invitations to complete the web-based questionnaire were disseminated at university events and activities, staff and student electronic newsletters, and through promotion by representative bodies including the University College Union and Guild of Students (Student’s Union). Data collection performed from May 2016 to April 2017 when the online survey was closed.

### Participants

Eligible study participants included all directly employed staff and undergraduate/postgraduate students enrolled on courses at the Edgbaston Campus, University of Birmingham during the data collection period (Total *N* = ~ 38,000 persons).

### Measures

#### Demographic variables

All respondents were invited to provide their age, sex, ethnic group and current university role (staff or student status). University staff were classified by staff employment group (professional services/academic staff) and students by degree level (undergraduate/postgraduate), and fee status (home/EU/international).

#### Tobacco usage, intention for smoking cessation and SHS exposure

Survey respondents were asked to provide their tobacco smoking status (*current smoker/previous smoker/never smoker*) and those who reported current smoking activity were sub-classified into *daily smokers* (tobacco smoking on a daily basis) or *intermittent smokers* (tobacco smoking less than daily)*.* Among *current* and *previous smokers*, information was obtained regarding tobacco smoking or use of e-cigarettes/vaping devices on the university campus and type(s) of tobacco products consumed (manufactured cigarettes, hand-rolled cigarettes, tobacco pipes, cigars, water or shisha pipe, e-cigarettes). Participants who reported being *current* or *previous smokers*, also responded to questions regarding smoking cessation, including current *intention to quit* status, defined as current intention to quit smoking or a quit attempt within the past 12 months.

#### Attitudes and support towards a campus tobacco control policy

Items reported in the study concerning staff and student’s attitudes and levels of support for specific policy options were obtained from the 21-item survey questionnaire. Questions related to aspirations for a tobacco or smoke-free campus, provision of smoking cessation services and level of support for no-smoking signage and smoking shelters. A Likert scale was used to assess level of agreement with statements, with response options ranging from 1 (*strongly disagree*) to 5 (*strongly agree*). Binary variables were created to measure agreement with selected statements, with values 0 (*strongly disagree/disagree/unsure*) and 1 (*agree/strongly agree*). Two dichotomous variables were created to reflect our selected policy definitions: (i) *smoke-free campus policy support* - coded as ‘1’ for those respondents providing a response of *agree/strongly agree* to the statement concerning *an aspiration for a smoke-free campus*; (ii) *comprehensive tobacco-free campus policy support -* coded as ‘1’ for those respondents who provided a response of *agree/strongly agree* for all three statements concerning: (a) *an aspiration for smoke-free campus,* (b) *restrictions for e-cigarettes/vaping on campus*, (c) *a ban of tobacco sales on campus.*

### Statistical analysis

Descriptive statistics including means, proportions (%) and corresponding 95% confidence intervals (95% CI) were calculated to summarise key demographic variables. Prevalence ratios (PRs) were calculated to evaluate comparisons between smoking status by demographic characteristics (gender, ethnicity) and staff/student status, with differences evaluated by Chi-square tests, with *P* ≤ 0.05 considered statistically significant. Logistic regression analyses was conducted to calculate odds ratios (OR) to report associations between participant characteristics and support for a smoke-free or comprehensive tobacco-free campus policy, after controlling for confounding factors. All statistical analyses were performed in Stata v13 (StataCorp, US).

### Ethical approval

Ethical approval for the study was provided by the University of Birmingham Research Ethics Committee (Ref ERN_16–0409). Confidentiality was assured for all participants and no identifiable information was collected from respondents. The survey did not include financial or other incentives for participation.

## Results

A total of 934 survey responses were received (estimated response rate 2.5%), of whom 93.6% (*N* = 874) provided consent for information to be used for research purposes. Those respondents who provided information regarding staff or student status (*N* = 780) were included in the present analysis (Table 1). The majority of participants were university staff (69.9%, *n* = 545), most of whom were from professional services (72.4%, *n* = 394) with a lower proportion of academic staff (20.6%, *n* = 112). Just under one third of survey respondents were university students (30.1%, *n* = 235), with most studying at undergraduate level (84.3% *n* = 198). Among participants included in the analysis, 59.6% (*n* = 465) were females and 39.5% (*n* = 308) males, and 86% (*n* = 657) identified themselves as of White British/Irish ethnicity. The mean age was 42 years (SD 11.4) and 22 years (SD 11.4), for staff and students respectively.

**Table 1:** Demographic characteristics of survey participants (university staff and students)

Demographic Characteristics	Staff *N* = 545 n (%)	Students *N* = 235 n (%)	Total *N* = 780
Age			
17–24 years	25 (4.8)	198 (85.7)	223 (29.7)
25–34 years	131 (25.2)	22 (9.5)	153 (20.4)
35–44 years	150 (28.9)	9 (3.9)	159 (21.2)
45–54 years	123 (23.7)	2 (0.9)	125 (16.6)
≥ 55 years	91 (17.5)	0 (0.0)	91 (12.1)
Gender			
Male	222 (41.1)	86 (36.9)	308 (39.8)
Female	318 (58.9)	147 (63.1)	465 (60.2)
Ethnic Group			
White British/Irish	493 (92.5)	164 (71.0)	657 (86.0)
Mixed/Multiple	12 (2.3)	14 (6.1)	26 (3.4)
Asian/Asian British	15 (2.8)	27 (11.7)	42 (5.5)
Black African/Caribbean	6 (1.1)	11 (4.8)	17 (2.2)
Other ethnic group	7 (1.3)	15 (6.5)	22 (2.9)
University Role (Staff only)			
Academic Staff	112 (20.6)	–	
Professional Services Staff	394 (72.4)	–	
Other Staff	38 (7.0)	–	
Degree Status (students only)			
Undergraduate Student	–	198 (84.3)	
Postgraduate Student	–	37 (15.7)	
Fee Status (students only)			
Home	–	186 (80.2)	
EU	–	24 (10.3)	
International	–	22 (9.5)	
Smoking Status			
Never Smoker	337 (62.1)	171 (73.7)	511 (65.7)
Previous Smoker	133 (24.5)	25 (10.8)	158 (20.3)
Intermittent Smoker	15 (2.8)	16 (6.9)	31 (4.0)
Daily Smoker	58 (10.7)	25 (10.8)	78 (10.1)

Missing data: age (29), gender (7), ethnic group (16), university role (1), fee status (3), smoking status (2)

### Tobacco smoking and usage patterns

Prevalence of current tobacco smoking (daily or intermittent) was 14.0% (*n* = 109; 95% CI: 11.6–16.6%), with no significant difference between university staff and students (13.4% vs 15.5%, *P* = 0.48). The proportion of current smokers was higher among males compared to females (19.0% vs. 11.0%, *P* = 0.002) (Additional file 1: Table S1). Overall, 34% (95% CI: 30.8–37.6%) of participants were former smokers, with the highest prevalence among males aged 45–54 years (40.0%). There was a higher prevalence of *previous smoking* among males (PR: 1.25, *P* = 0.037), staff members (PR: 1.44, *P* = 0.003) and those of White British/Irish ethnicity (PR: 1.54, *P* = 0.005). Almost one half (49.5%, *n* = 53) of current tobacco smokers reported they wished to quit smoking and almost one third (31.5%, *n* = 34) had attempted to quit within the previous 12 months. Among current smokers (*n* = 109), the majority (90.8%, *n* = 99) had smoked on campus, and the predominant tobacco product choice was manufactured (45.4%) or hand-rolled cigarettes (38.9%), with 8.3%, reporting ever to have used e-cigarettes on campus and a small number (7.4%, *n* = 8) other tobacco products (e.g. cigars, shisha, hookah) (data not shown).

### Attitudes and support towards a tobacco control policy

Overall, 86.8% (95% CI: 84.2–89.1%) of survey respondents agreed that staff and students should not be exposed to SHS on campus, 66.3% (95% CI: 62.9–69.7%) supported an aspiration for a smoke-free university campus, and 68.5% (95% CI: 65.2–71.8%) endorsed restrictions for tobacco sales. In both staff and student samples, support for a smoke-free campus was strongly associated with smoking status; the highest level of support was among non-smoking students (80.6%; 95% CI: 74.4–85.9%) and staff members (72.6%; 95% CI: 68.3–76.6%) respectively (Table 2). Just under half of respondents (47.3%; 95% CI: 43.8–50.9%) supported a ban for e-cigarettes/vaping device use on campus, with significant differences by smoking status. Support for smoking cessation provision was higher among students (94.9%; 95% CI: 89.6–96.8%) compared to staff members (86.1%; 95% CI: 70.5–95.3), with the majority of respondents in both groups favouring smoking shelter and no-smoking signage provision. The majority of current smokers (90.8%) felt that a comprehensive tobacco-free campus would discriminate against and disadvantage staff and students who smoke, with fewer non-smokers considering it would be a discriminative policy, among both staff (34.6%; 95% CI: 30.3–39.2%) and student (28.3%; 95% CI: 21.9–34.9%) groups respectively. The majority of participants reported that a smoke-free campus policy would improve the health of staff and students (staff 80.6%; students 89.3%) and the University’s public image (staff: 67.2%; students 81.3%).

**Table 2:** Tobacco control policy support and perceptions among university staff and students

	Staff				Students			
	Current Smokers		Non-Smokers		Current Smokers		Non-Smokers	
	*N* = 73	% (95% CI)	*N* = 470	% (95% CI)	*N* = 36	95% CI	*N* = 196	95% CI
Support for a campus-wide tobacco control policy								
*Staff and students should not be exposed to Second Hand Smoke on campus*	43	59.7 (47.5–71.1)	417	88.9 (85.7–91.6)	22	61.1 (43.5–76.9)	189	96.4 (92.8–98.6)
*We should aspire to make the university free of tobacco smoking*	11	15.3 (7.9–25.7)	342	72.6 (68.3–76.6)	4	11.1 (3.1–26.1)	158	80.6 (74.4–85.9)
*Staff and students should not be allowed to smoke e-cigarettes on campus*	10	13.7 (6.8–23.8)	248	52.6 (47.9–57.1)	6	16.7 (6.4–32.8)	104	53.1 (45.8–60.2)
*Tobacco sales should be banned on campus*	12	16.4 (8.8–27.0)	345	73.9 (69.6–77.8)	9	25.0 (12.1–42.2)	165	83.2 (77.2–88.1)
Support for tobacco control policy intervention measures								
*Stop smoking support should be provided on campus*	49	67.1 (55.1–77.7)	383	82.0 (78.2–85.4)	33	91.7 (77.5–98.2)	180	92.8 (88.2–96.0)
*Smoking shelters should be provided on campus*	57	78.1 (66.9–86.9)	183	39.3 (34.8–43.9)	29	80.6 (64.0–91.8)	98	50.0 (42.8–57.2)
*No-smoking signs should be clearly placed around campus*	45	63.4 (51.1–74.5)	408	87.7 (84.4–90.6)	18	51.4 (34.0–68.6)	176	90.3 (85.3–94.0)
Perceptions of a campus-wide tobacco control policy								
*A tobacco-free campus would improve the University’s public image*	11	15.1 (7.7–25.4)	323	69.0 (64.6–73.2)	8	22.2 (10.1–39.1)	165	84.2 (78.2–89.0)
*A tobacco-free campus would improve the health of staff and students*	23	31.5 (21.1–43.4)	389	83.5 (79.8–86.7)	15	41.7 (25.5–59.2)	182	93.3 (88.9–96.4)

Table 3 displays the regression analyses to identify independent predictors of support for smoke-free and comprehensive tobacco-free university campus policies respectively. The strongest predictive factor was smoking status, with likelihood of support for both a smoke-free or tobacco-free campus significantly lower among daily smokers (Adjusted OR (AOR) 0.02, 95% CI: 0.01–0.05 and AOR 0.02, 95% CI: 0.00–0.10) compared to never smokers (*P* < 0.001) after adjustment for measured confounding factors. Support was also lower among previous smokers compared to never smokers for both smoke-free (AOR 0.28; 95% CI: 0.18–0.42) or tobacco-free policy (AOR 0.39; 95% CI: 0.26–0.60) policies respectively. Support for a smoke-free campus policy was also significantly more likely among females (AOR 1.45; 95% CI: 1.00–2.11) and those of Asian/Asian British ethnicity (AOR 5.46, 95% CI: 1.49–19.96), who were also more likely to support a comprehensive tobacco-free campus policy (AOR 2.07, 95% CI: 1.02–4.20). There were no significant observed differences in support level by university role (staff/student) or age group in adjusted analyses.

**Table 3:** Logistic regression analyses reporting support for (i) smoke-free and (ii) tobacco-free campus policy options among university staff and students

	Smoke-Free Campus^a^				Tobacco-Free Campus^b^		
Predictor variable	N	Adjusted OR	95% CI	*P*-value†	Adjusted OR	95% CI	*P*-value
Age							
17–24 years	223	–	–		–	–	
25–34 years	153	0.73	0.34–1.52	0.394	0.81	0.42–1.56	0.521
35–44 years	159	1.54	0.68–3.48	0.297	1.45	0.72–2.92	0.301
45–54 years	125	0.70	0.31–1.60	0.396	1.26	0.60–2.65	0.547
≥ 55 years	91	0.96	0.40–2.30	0.928	1.41	0.65–3.04	0.382
Gender							
Male	308	–	–		–	–	
Female	465	1.45	1.00–2.11	0.048*	0.99	0.71–1.38	0.932
Ethnic Group							
White British/Irish	657	–	–		–	–	
Mixed/Multiple	26	1.55	0.52–4.55	0.452	1.45	0.59–3.58	0.413
Asian/Asian British	42	5.46	1.49–19.96	0.010*	2.07	1.02–4.20	0.043*
Black African/Caribbean	17	0.88	0.25–3.07	0.800	1.18	0.43–3.24	0.742
Other ethnic group	22	0.98	0.33–2.96	0.974	2.39	0.93–6.11	0.069
University Status							
Student	233						
Staff	544	1.11	0.54–2.27	0.810	1.08	0.56–2.02	0.844
			–		–	–	
Smoking Status							
Never Smoker	508	–	–	–	–	–	
Previous Smoker	158	0.28	0.18–0.42	< 0.001**	0.39	0.26–0.60	< 0.001**
Intermittent Smoker	31	0.06	0.02–0.16	< 0.001**	0.13	0.04–0.43	0.001**
Daily Smoker	78	0.02	0.01–0.05	< 0.001**	0.02	0.00–0.10	< 0.001**

†*P*-value for differences between groups *P ≤ 0.05 **P ≤ 0.001

^a^Agreement/strong agreement with the survey item: *‘We should aspire to make the university campus free of tobacco smoking’*

^b^Agreement/strong agreement with all survey items: *(a) We should aspire to make the university campus free of tobacco smoking’;* and *(b) Tobacco sales should be banned on campus’;* and *(c) Staff and students should not be allowed to smoke e-cigarettes on campus*

## Discussion

This study provides insights into the contemporary smoking behaviours and attitudes towards smoke and comprehensive tobacco-free policy options, among 780 staff and students attending a large UK University. Overall, smoking prevalence was 14.0% (95% CI 11.6–16.6%) and over two-thirds of respondents (68.5%; 95% CI: 65.2–71.8%) expressed support for a smoke-free campus and just under half (47.3%; 95% CI: 43.8–50.9%) support a ban for e-cigarettes/vaping device use on campus. Smoking status was an independent predictor of support for tobacco control, with the lowest level of support for a smoke-free campus among daily and intermittent smokers. Our novel findings provide valuable baseline information regarding patterns of smoking within a university campus environment, which may inform development and enable future evaluation of a revised voluntary campus-based tobacco control policy option in the context of a UK higher education institution.

Prevalence of current tobacco smoking (14.0%) was marginally lower than the UK adult population (15.1%), [23] but broadly consistent with smoking rates observed in postsecondary educational settings in the United States and New Zealand [24, 25]. The proportion of current smokers reporting an intention to quit was lower than the national average (49.5% vs 60.8%) [26] potentially reflecting the demographic characteristics of our study population. Further differences were observed in e-cigarette usage patterns, with current usage reported by (8.3%) which is lower than the proportion of UK adult population who have tried an e-cigarette (19.4%), but higher than the proportion of current users in a national context (5.5%) [26].

Support for a campus-wide smoke-free policy was consistently high among both staff and students, with 86% of respondents expressing concern about SHS exposure and two-thirds (66.3%) supportive of an aspiration for a smoke-free campus. Attitudes towards inclusion of e-cigarettes or vaping devices within a smoke-free policy were less consistent; potentially due to mixed public awareness of the health impacts associated with vapour from these sources, or their role in supporting a smoke-free environment. Our findings are notably consistent with those of a meta-analysis of 19 studies performed by Lupton and colleagues, which found 58.9% of students and 68.4% of staff to be supportive of smoke-free campus policies [27]. Similar levels of support were observed within a cross-sectional survey at Curtin University, Western Australia, where 84.1% of respondents were concerned about the harms of SHS exposure and 65.7% supportive of a smoke-free campus policy option, with comparable differences by smoking status [20].

The majority of participants reported that a smoke-free campus policy would have a positive impact upon the health of staff and students, suggesting awareness of the links between smoking and tobacco usage and associated health harms. Although we did not seek specific views on the impact of a revised policy upon quality of life measures, given attitudes towards a smoke-free campus were broadly positive, such an association suggests potential to achieve wider improvements in staff and student wellbeing. Raising awareness of relevant health messages and reinforcement of the harms of SHS exposure are likely to improve acceptance and policy compliance, as previously observed in bar and restaurant settings [28].

Understanding the factors associated with support among population sub-groups may be beneficial for leveraging relevant support and promotion of positive attitudes towards change. Consistent with other investigators, [29] we observed a gradient across categories of smoking status, with the lowest level of policy endorsement among daily, compared to intermittent and former smokers, and highest among never smokers. These attitudes may be magnified by concern around stigma, reflected in the high proportion of smokers (90.4%), who considered a smoke-free policy to be discriminative [29]. Poland and colleagues (2012) [30] described the importance of characterisation of discrete types of smokers to inform targeted mitigation measures, identifying that ‘easygoing’ smokers were supportive of smoking restrictions if implemented sensitively and supported with appropriate messages.

In accordance with best practice in health promotion theory, [31] a comprehensive range of strategies including support for current smokers is most likely to achieve optimal outcomes. This assumption is further supported by existing evidence for workplace smoking restrictions as motivators for behaviour change; underpinned by the relatively high proportion of survey participants within the contemplative phase of health behaviour change [32] (intention to quit or quit attempt), suggesting policy implementation is likely to be most effective if integrated with smoking cessation provision.

This study had a number of strengths and limitations. Although the overall survey response rate was relatively low, the large study population comprises a diverse cohort of university staff and students. Males were slightly underrepresented comprising only 39.8% of participants, as were EU (10.3%) and international students (9.5%); however, this response pattern is similar to other campus smoking studies [18]. We did not assess income or composite measures of socio-economic status, which are potential confounding factors; however, information was available for age, sex, ethnic group and staff status. The element of selective non-response bias may have resulted in more positive attitudes towards tobacco control policies than among the total university population; however, with the sample size of 780, our findings provide the most comprehensive information available concerning contemporary smoking behaviours and attitudes in a UK tertiary educational setting.

Use of a self-administered questionnaire provides only a subjective assessment of smoking status, and could be influenced by social acceptability bias; however, we did not collect identifiable information and participants were able to exclude their responses from research purposes. Our survey did not include questions regarding symptoms of smoke related illness or awareness of the harms of SHS or Thirdhand Smoke (THS) exposure, which may be better explored through future qualitative research*.* We administrated the questionnaire at a single time point, yet plan to conduct a repeat cross-sectional survey at a future date to explore changes in prevalence, attitudes and levels of support over time [16].

### Implications for policy and research

The WHO FCTC suggests that national bodies and organisations should protect the population from hazards of SHS ‘wherever the evidence shows that hazard exists’, including quasi-outdoor and outdoor places [3]. Despite gaining popularity worldwide, there remains limited research regarding attitudes towards and effectiveness of smoke- and tobacco-free campus policies. However, it is widely recognised that achieving effective adoption of smoke-free legislation in any setting requires population support and a high degree of compliance.

Potential challenges in local policy implementation include enforcement difficulties, smoking displacement, self-perceived workplace stress, negative community relations and safety concerns [20]; however, relevant mitigation measures may include phased smoke-free zones or designated shelter provision. These processes will require robust future implementation research, to develop the evidence base concerning policy implementation and organisational change processes, to inform widespread adoption of smoke-free and comprehensive tobacco-free policies across UK higher education institutions.

## Conclusion

Our findings indicate that the majority of staff and students at the University of Birmingham broadly support introduction of a campus-wide smoke-free or comprehensive tobacco-free policy. Provision of a package of supporting measures including smoking cessation support and smoking shelters may improve policy implementation and compliance. Further research to improve our current understanding of social and organisational norms which might influence policy adoption and compliance, including exploration of preferences and priorities among specific population sub-groups, will help inform effective policy implementation.

## Supplementary information

**Additional file 1: Table S1.** Demographic characteristics of study participants by smoking status

## Data Availability

The datasets used and/or analysed during the current study are available from the corresponding author on reasonable request.

## Abbreviations

AOR: Adjusted odds ratio
E-cigarette: Electronic cigarette
HSE: Health survey for England
OR: Odds ratio
PR: Prevalence ratio
SHS: Secondhand smoke
THS: Thirdhand smoke
WHO-FCTC: World Health Organization Framework Convention for Tobacco Control

## References

[1] Digital NHS (2018). Statistics on smoking - England, 2019.

[2] U.S. Department of Health and Human Services (2014). The health consequences of smoking – 50 years of progress: a report of the Surgeon General. Centers for Disease Control and Prevention NCfCDPaHP, Office on Smoking, Health a, editors. Atlanta.

[3] Chung-Hall J, Craig L, Gravely S, Sansone N, Fong GT (2019). Impact of the WHO FCTC over the first decade: a global evidence review prepared for the impact assessment expert group. Tob Control.

[4] Frazer K, Callinan JE, McHugh J, van Baarsel S, Clarke A, Doherty K (2016). Legislative smoking bans for reducing harms from secondhand smoke exposure, smoking prevalence and tobacco consumption. Cochrane Database Syst Rev.

[5] Center for Disease Control and Prevention (2007). Reduced secondhand smoke exposure after implementation of a comprehensive statewide smoking ban--New York, June 26, 2003-June 30, 2004. MMWR Morb Mortal Wkly Rep.

[6] Department for Education (2017). Participation Rates In Higher Education: Academic Years 2006/2007–2015/2016 (Provisional).

[7] Eckhardt L, Woodruff SI, Elder JP (1994). A longitudinal analysis of adolescent smoking and its correlates. J Sch Health.

[8] Ickes MJ, Rayens MK, Wiggins A, Hahn EJ (2017). Students’ beliefs about and perceived effectiveness of a tobacco-free campus policy. Policy Polit Nurs Pract.

[9] Wolfson M, McCoy TP, Sutfin EL (2009). College students’ exposure to secondhand smoke. Nicotine Tob Res.

[10] Peto R, Darby S, Deo H, Silcocks P, Whitley E, Doll R (2000). Smoking, smoking cessation, and lung cancer in the UK since 1950: combination of national statistics with two case-control studies. BMJ.

[11] Gerson M, Allard JL, Towvim LG (2005). Impact of smoke-free residence hall policies: the views of administrators at 3 state universities. J Am Coll Heal.

[12] Wang TW, Tynan MA, Hallett C, Walpert L, Hopkins M, Konter D (2018). Smoke-free and tobacco-free policies in colleges and universities - United States and territories, 2017. MMWR Morb Mortal Wkly Rep.

[13] Trad C, Bayly J, Saint-Fort L, Andrews M, Patel M, Sabado-Liwag M (2018). Adoption of tobacco- and smoke-free policies in a US National Sample of postsecondary educational institutions. Am J Public Health.

[14] Seo DC, Macy JT, Torabi MR, Middlestadt SE (2011). The effect of a smoke-free campus policy on college students’ smoking behaviors and attitudes. Prev Med.

[15] Lee JG, Ranney LM, Goldstein AO (2013). Cigarette butts near building entrances: what is the impact of smoke-free college campus policies?. Tob Control.

[16] Lechner WV, Meier E, Miller MB, Wiener JL, Fils-Aime Y (2012). Changes in smoking prevalence, attitudes, and beliefs over 4 years following a campus-wide anti-tobacco intervention. J Am Coll Heal.

[17] Fallin A, Roditis M, Glantz SA (2015). Association of campus tobacco policies with secondhand smoke exposure, intention to smoke on campus, and attitudes about outdoor smoking restrictions. Am J Public Health.

[18] Satterlund TD, Cassady D, Treiber J, Lemp C (2011). Barriers to adopting and implementing local-level tobacco control policies. J Community Health.

[19] Braverman MT, Hoogesteger LA, Johnson JA, Aaro LE (2017). Supportive of a smoke-free campus but opposed to a 100% tobacco-free campus: identification of predictors among university students, faculty, and staff. Prev Med.

[20] Burns S, Bowser N, Smith J, Jancey J, Crawford G (2014). An exploratory study of smokers’ and stakeholders’ expectations of the implementation of a smoke-free policy in a university setting. Health Promot J Austr.

[21] Giovino GA, Mirza SA, Samet JM, Gupta PC, Jarvis MJ, Bhala N (2012). Tobacco use in 3 billion individuals from 16 countries: an analysis of nationally representative cross-sectional household surveys. Lancet.

[22] Health Survey for England (HSE) London: University College London; 2016 [Available from: https://www.ucl.ac.uk/hssrg/studies/hse.

[23] Office for National Statistics (2017). Adult smoking habits in the UK.

[24] Marsh L, Robertson LA, Cameron C (2014). Attitudes towards smokefree campus policies in New Zealand. N Z Med J.

[25] Kypri K, Baxter J (2004). Smoking in a New Zealand university student sample. N Z Med J.

[26] Office for National Statistics (2018). E-cigarette use in Great Britain.

[27] Lupton JR, Townsend JL (2015). A systematic review and meta-analysis of the acceptability and effectiveness of university smoke-free policies. J Am Coll Heal.

[28] Borland R, Yong HH, Siahpush M, Hyland A, Campbell S, Hastings G (2006). Support for and reported compliance with smoke-free restaurants and bars by smokers in four countries: findings from the International Tobacco Control (ITC) Four Country Survey. Tob Control.

[29] Rigotti NA, Regan S, Moran SE, Wechsler H (2003). Students' opinion of tobacco control policies recommended for US colleges: a national survey. Tob Control.

[30] Poland BD, Cohen JE, Ashley MJ, Adlaf E, Ferrence R, Pederson LL (2000). Heterogeneity among smokers and non-smokers in attitudes and behaviour regarding smoking and smoking restrictions. Tob Control.

[31] Howat P, Maycock B, Cross D, Collins J, Jackson L, Burns S (2003). Towards a more unified definition of health promotion. Health Promotion J Australia.

[32] Prochaska JO, Redding CA, Evers KE, Glanz K, Rimer BK, Viswanath K (2015). The Transtheoretical Model and Stages of Change. Health Behavior: Theory, Research, and Practice.

