# Supplementary material for: Smoking behaviours and attitudes towards campus-wide tobacco control policies among staff and students: a cross-sectional survey at the University of Birmingham
Source: BMC Public Health. 2020 Feb 19;20:252. doi: 10.1186/s12889-020-8321-9 (PMC7031967; doi:10.1186/s12889-020-8321-9)
Supplement: Supplementary file 1 — Additional file 1: Table S1. Demographic characteristics of study participants by smoking status [file 12889_2020_8321_MOESM1_ESM.docx]

**Table S1.** Demographic characteristics of study participants by smoking status

|  | N | Previous Smoker  n (%) | *P*-value | Current smoker  n (%) | *P*-value† |
| --- | --- | --- | --- | --- | --- |
| Age |  |  |  |  |  |
| 17 -24 years | 223 | 54 (24.3) | P=0.004* | 29 (13.1) | *P*=0.164 |
| 25 – 34 years | 153 | 55 (36.4) |  | 23 (15.0) |  |
| 35-44 years | 159 | 65 (41.1) |  | 26 (16.5) |  |
| 45 – 54 years | 125 | 50 (40.0) |  | 22 (17.6) |  |
| ≥55 years | 91 | 30 (33.0) |  | 6 (6.6) |  |
| Gender |  |  |  |  |  |
| Male | 308 | 118 (38.8) | *P*=0.037* | 58 (19.0) | *P*=0.002* |
| Female | 465 | 144 (31.0) |  | 51 (11.0) |  |
| Ethnic Group |  |  |  |  |  |
| White British/Irish | 657 | 242 (36.9) | *P*=0.005* | 91 (14.1) | *P*=0.654 |
| Mixed/Multiple ethnic | 26 | 7 (28.0) |  | 4 (16.0) |  |
| Asian/Asian British | 42 | 8 (19.5) |  | 4 (9.5) |  |
| Black/African/Caribbean | 17 | 1 (5.9) |  | 1 (5.9) |  |
| Other ethnic group | 22 | 4 (18.2) |  | 2 (9.1) |  |
| University Role |  |  |  |  |  |
| Undergraduate Student | 196 | 48 (24.6) | *P*=0.003* | 28 (14.4) | *P*=0.349 |
| Postgraduate Student | 37 | 13 (35.1) |  | 8 (21.6) |  |
| Academic Staff | 112 | 32 (29.1) |  | 12 (10.7) |  |
| Professional Services Staff | 394 | 159 (40.4) |  | 53 (13.5) |  |
| Other Staff | 38 | 13 (35.1) |  | 8 (21.1) |  |

†*P*-value for differences in smoking prevalence between groups *≤0.05 **≤0.001
